# Innervation: the missing link for biofabricated tissues and organs

**DOI:** 10.1038/s41536-020-0096-1

**Published:** 2020-06-05

**Authors:** Suradip Das, Wisberty J. Gordián-Vélez, Harry C. Ledebur, Foteini Mourkioti, Panteleimon Rompolas, H. Isaac Chen, Mijail D. Serruya, D. Kacy Cullen

**Affiliations:** 1 Center for Brain Injury & Repair, Department of Neurosurgery, Perelman School of Medicine, University of Pennsylvania, Philadelphia, PA USA; 2 Center for Neurotrauma, Neurodegeneration & Restoration, Corporal Michael J. Crescenz Veterans Affairs Medical Center, Philadelphia, PA USA; 3 Department of Bioengineering, School of Engineering and Applied Science, University of Pennsylvania, Philadelphia, PA USA; 4 Axonova Medical, LLC., Philadelphia, PA USA; 5 Department of Orthopedic Surgery, Perelman School of Medicine, University of Pennsylvania, Philadelphia, PA USA; 6 Department of Cell and Developmental Biology, Perelman School of Medicine, University of Pennsylvania, Philadelphia, PA USA; 7 Department of Dermatology, Perelman School of Medicine, University of Pennsylvania, Philadelphia, PA USA; 8 Department of Neurology, Thomas Jefferson University, Philadelphia, PA USA

**Keywords:** Tissue engineering, Regenerative medicine

## Abstract

Innervation plays a pivotal role as a driver of tissue and organ development as well as a means for their functional control and modulation. Therefore, innervation should be carefully considered throughout the process of biofabrication of engineered tissues and organs. Unfortunately, innervation has generally been overlooked in most non-neural tissue engineering applications, in part due to the intrinsic complexity of building organs containing heterogeneous native cell types and structures. To achieve proper innervation of engineered tissues and organs, specific host axon populations typically need to be precisely driven to appropriate location(s) within the construct, often over long distances. As such, neural tissue engineering and/or axon guidance strategies should be a necessary adjunct to most organogenesis endeavors across multiple tissue and organ systems. To address this challenge, our team is actively building axon-based “living scaffolds” that may physically wire in during organ development in bioreactors and/or serve as a substrate to effectively drive targeted long-distance growth and integration of host axons after implantation. This article reviews the neuroanatomy and the role of innervation in the functional regulation of cardiac, skeletal, and smooth muscle tissue and highlights potential strategies to promote innervation of biofabricated engineered muscles, as well as the use of “living scaffolds” in this endeavor for both in vitro and in vivo applications. We assert that innervation should be included as a necessary component for tissue and organ biofabrication, and that strategies to orchestrate host axonal integration are advantageous to ensure proper function, tolerance, assimilation, and bio-regulation with the recipient post-implant.

## Introduction

The nervous system consists of the brain and the spinal cord, jointly known as the central nervous system (CNS), and the rest of the nerves of the body that constitute the peripheral nervous system (PNS)^1^. In addition to higher-order function, the CNS is involved in integrating sensory information to indirectly coordinate the functions of other tissues and organs. This regulation is executed through the connections between the CNS and the nerves of the PNS that directly interact with the rest of the body. The primary mediators of these nerve–organ connections are axons, which are fibrous projections from nerve cells, called neurons. These axonal connections mediate precise junctions with the end target, and enable the critical role the nervous system has in the development, maturation, function, regulatory control, and, indeed, even regeneration and pathology of tissues and organs. The primary involuntary (i.e., not requiring conscious thought) component of these nerve–organ interactions is referred to as the autonomic nervous system (ANS), which is involved in the regulation of tissues and organs through the parasympathetic, sympathetic, and enteric components^2^. Parasympathetic and sympathetic nerves emanate from the spinal cord and synapse with ganglia from which postganglionic fibers reach their targets to regulate rest and stress responses, respectively, in coordination with the CNS^3^. The enteric nervous system (ENS) is intrinsic to the gastrointestinal (GI) tract, innervating the gut wall and acting fairly independently; although the ENS can still be influenced by parasympathetic and sympathetic action^4^. On the other hand, the somatic nervous system (SNS) deals with voluntary control of skeletal muscle through motor axons and transmission of information to the CNS through sensory axons^3^.

Normal tissue and organ innervation can be altered, in a partial or complete manner, due to surgery, trauma, and neurological disease. For instance, corrective procedures such as congenital heart surgery in infants can disrupt sympathetic innervation, thereby increasing the risk of sudden cardiac arrest later in adult life^5^. Patients with spinal cord injury (SCI), peripheral nerve injury (PNI), and amyotrophic lateral sclerosis (ALS) suffer functional limitations associated with muscle denervation and atrophy^6^. Organ transplant surgeries often result in the ablation of autonomic connections, which may cause poor functionality and detrimental health effects. Moreover, there is a critical need for alternative transplant strategies to address tissue and organ failure other than allografts and autografts due to the overwhelming shortage of donor tissue, failure to adequately track and address allograft rejection, problems with donor site morbidity and long-term stability, long-term administration of immunosuppressants, and possible side effects of these drugs^7–9^. Due to the significance of peripheral innervation, restoring proper axonal integration with tissues and organs affected by trauma or disease and ensuring that implanted substitutes successfully integrate with the host nervous system are goals in dire need of attention and action.

A particularly noteworthy area is the search for alternative strategies for the replacement of tissues and organs containing cardiac, skeletal, or smooth muscle cells, given their ubiquitous presence in so many apparent and non-apparent basic life functions and the immense burden associated with diseases and trauma related to these muscles. For example, cardiovascular disease was responsible for 31% of worldwide deaths in 2016^10^. Heart transplants from a deceased donor are currently the gold standard treatment option for end-stage heart failure, a condition in which the heart inefficiently meets the body’s demand for blood flow and that afflicts ~6.5 million people in the United States over the age of 20^11^. Moreover, cardiac tissue has an extremely limited capacity for regeneration, reinforcing the need for other treatment options. In the case of skeletal muscle, while there is generally more capability for regeneration, this intrinsic ability is insufficient in the case of volumetric muscle loss (VML), where the injury is such that cells responsible for regeneration are lost, leading to fibrotic scar tissue formation, severe functional deficits, and impaired quality of life^12^. Highlighting the far-reaching applicability of new putative interventions, significant muscle loss can result from a variety of situations including traffic accidents, sports and combat injuries, surgeries, and genetic diseases, among others. Furthermore, smooth muscle tissue is a component of many tissues and organs, forming the muscle layers in the GI wall that allow for intestinal motility, the bladder wall to store and release urine, and the walls of blood vessels having a role in vasoconstriction and vasodilation, among others^3^. Given the abundance of smooth muscle, this tissue type is of high relevance in any strategy attempting to reconstruct or replace damaged segments of the GI tract, the bladder, and blood vessels in myriad diseases. In these endeavors in cardiac, skeletal, and smooth muscle replacement, there has been, at best, an inconsistent focus on also promoting (re)innervation even if nerve fibers have a pivotal role in their ultimate function within the body.

**Fig. 1:**
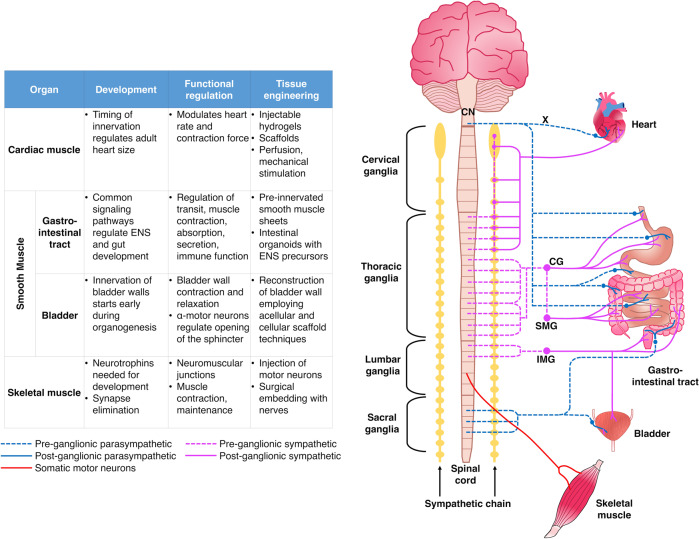
Innervation of cardiac, skeletal, and smooth muscle. The table (left) presents a summary of innervation in the development and functional regulation of the heart (cardiac muscle), GI tract and bladder (smooth muscle), and skeletal muscle, and tissue engineering approaches for these tissues and cases where innervation has been considered in these strategies. The schematic (right) presents parasympathetic and sympathetic inputs to the organs surveyed in this manuscript; only connections with one side are shown for simplicity. We also show somatic fibers connected with skeletal muscle (bottom right). Preganglionic parasympathetic fibers emanate mostly from the brainstem, except for innervation to the lower large intestine and the bladder, which originate from the sacral spinal cord. Most preganglionic parasympathetic nerves synapse with ganglia in close proximity to the target organ (shown as blue circles near or at the organs). On the other hand, preganglionic sympathetic fibers exit from the gray matter in the thoracic or lumbar spinal cord and interact with ganglia in the bilateral sympathetic chain (yellow). In other cases, preganglionic fibers within the splanchnic nerves pass through the sympathetic chain to synapse further on with abdominal ganglia. *CN* cranial nerve, *CG* celiac ganglia, *SMG* superior mesenteric ganglion, *IMG* inferior mesenteric ganglion.

In this review article, we propose addressing the challenge of innervation in tissue replacement by fabricating artificial tissues and organs using biomaterials and tissue engineering techniques. Given the limited consideration for innervation in tissue engineering, we initially present innervation within the context of challenges in the field and the reasons why incorporating strategies to promote innervation would be beneficial. Then, we consider the examples of cardiac, skeletal, and smooth muscle and survey the anatomy of their innervation, implications of innervation in the functioning of tissue-engineered muscles, and the current state of innervated muscle constructs (Fig. 1). Afterwards, we highlight potential strategies for promoting or incorporating innervation in the biofabrication of tissues and organs. Among these strategies, we present our axon-based “living scaffold” technology, consisting of “stretch-grown” tissue-engineered nerve grafts (TENGs)^13–16^ and micro-tissue engineered neural networks (micro-TENNs)^17–21^, which may be applied as living, axon-based bridges and interfaces to innervate biofabricated tissues and organs. We conclude by surveying current advances and key issues related to the fabrication of artificial tissues and organs, recognizing the multifaceted and multidisciplinary nature of the challenges ahead for the field of tissue engineering. We assert that, while innervation is part of a complex set of challenges in tissue engineering, artificial organs will likely significantly benefit from embedded neural cells that ensure proper development and function and from scaffolds that facilitate host innervation post-transplant in a controlled, targeted manner.

## Relevance of innervation in tissue engineering and regenerative medicine

By using a combination of cells, biomaterials, and signaling molecules, tissue engineering has been applied to create biological substitutes for a variety of tissues such as kidney^22^, intestine^23^, skeletal muscle^24^, pancreas^25^, skin^26^, heart^27^, among others. Tissue-engineered constructs should ideally be fabricated to emulate the three-dimensional (3D) microarchitecture and the mechanical and biochemical cues of the tissue of interest to allow for the implant to structurally and functionally integrate with the body in a proper manner. In spite of the potential for engineered organs to replace allogeneic organ transplantation, there are still challenges that limit their applicability such as the need to mimic the organization and coordinated action of multiple cell types in tissue and to promote integration between the constructs and the host vasculature and nervous system^28,29^. Recapitulating the heterogeneity of tissue has been systematically addressed by developing co-culture systems where two or more cell types are combined to resemble native tissue, although further work is required to more specifically define the cell types, sources and densities, the scaffolds, and the culture conditions required for each application^9^. Vascularization has long been recognized as one of the most pressing challenges in tissue engineering as it is essential for the survival of larger tissues and their access to nutrient exchange and oxygen. There have been substantial studies on techniques to promote vascularization of engineered tissues (e.g., scaffolds with microvascular networks, delivery of growth factors)^30–32^. On the other hand, implanted constructs typically lack preformed neural networks and depend on host-induced innervation to integrate with the native nerve supply, as has been observed in previous efforts with engineered urinary bladders^33^, skin^34^, and intestine^35^. Even though there have been studies on the transplantation of nerve grafts into target organs^36^ and some incursions into fabricating pre-innervated engineered tissue^37^, this pursuit has not been widespread and, moreover, not enough focus has been awarded to promoting targeted and appropriate connections between the host nervous system and biofabricated tissues and organs^38^. This general lack of recognition for innervation in tissue engineering mainly stems from the overwhelming complexity of many of the tissues and organs under study. Moreover, there also needs to be greater knowledge and appreciation in the field about the importance of innervation in native tissue and organ function and the benefits for engineered constructs.

Innervation has a pivotal role as a means for the functional control and modulation of tissues and organs. For example, based on sensory information about the environmental and physiological conditions in organs, autonomic nerves regulate functions such as vasoconstriction, pancreatic secretions, urination, gut motility, and saliva production^39^. Integrating engineered tissues and conventional organ transplants with the host nervous system may thus ensure their proper performance and functional responsiveness, via biofeedback, to the host. Restoring innervation is particularly relevant given the functional effects of denervation after transplants. For example, nerve damage associated with corneal transplants has been linked to immune dysfunction through a decrease in levels of transforming growth factor-beta in the aqueous humor and thus has contributed to the rejection of subsequent corneal transplants^40,41^. In a different case, detachment of vagal pulmonary afferents during lung transplants, where total reinnervation has been deemed unlikely, has been related to increased sympathetic activity and concomitant increments in resting heart rate^42,43^. In the case of heart transplants, where reinnervation may occur in an inconsistent and partial manner, denervation has been related to a depletion in circulating catecholamines, reduced exercise capacity, increased resting heart rate, and disturbed regulation of nocturnal blood pressure^44^. Promoting innervation may also lead to better outcomes for patients, as suggested by studies that highlighted the relationship between reinnervation and improvements on heart rate and contractile function during exercise, blood flow regulation, and life outcomes in heart transplant-recipients^45^. Furthermore, functional reinnervation of the transplanted heart enables angina to occur during myocardial infarction (MI), which can be a lifesaving warning^46^.

Although not extensively studied, innervation is also increasingly being recognized as an essential component of organ development and regeneration^38,47–49^. For example, autonomic nerves contribute to organogenesis, wound healing, and tissue regrowth^50^ by preserving phenotypes and function in stem cell niches and presenting growth and transcription factors necessary for the maintenance of migrating cells in wounds^49,51,52^. There have been reports of consequential roles for nerves during development such as pancreatic sympathetic innervation influencing islet cytoarchitecture and functional maturation^48^ and parasympathetic innervation regulating tubulogenesis in the salivary gland^53^. Presenting innervation and the related signaling cues during in vitro biofabrication processes may thus result in more mature and biomimetic tissues recapitulating the structure and function of native tissues and organs. Moreover, in many instances blood vessels and nerves follow the same paths, and thus they are functionally coupled and their interactions can reciprocally promote regeneration, ingrowth, and integration^54,55^. Indeed, promoting innervation can directly contribute to revascularization as autonomic and sensory nerves can release neuropeptides (e.g., neuropeptide Y, calcitonin gene-related peptide-I) that promote angiogenesis^55^.

Innervation has to be considered as one more component towards the goal of creating engineered tissues that faithfully resemble the cell microenvironment and the structure and function in vivo. In this pursuit, intimate knowledge about neuroanatomy, the development of innervation, and nerve-based mechanisms of functional regulation in each organ will serve as the basis for tissue engineering strategies. In addition to being applied to drive innervation of artificial tissues in vitro and in vivo, neural tissue engineering may be used to restore or regenerate nerve supplies damaged after surgery, trauma, and disease. Further research may also advance the treatment of organ and systemic disease, particularly by creating more spatially specific and biocompatible ways of stimulating nerve activity to modulate organ function. In the following sections, we consider cardiac, skeletal, and smooth muscle tissue engineering as case studies to discuss the role of innervation in these tissues, the relevance of cross-disciplinary neuromuscular tissue engineering, and instances in which innervation has been considered in the biofabrication of artificial muscle tissue.

## Innervation in cardiac muscle tissue engineering

The cardiac muscle or myocardium is composed of cardiomyocytes forming striated, tubular and branched muscle fibers under involuntary control. Cardiomyocytes are predominantly uninucleated in humans whereas in other mammals, such as rats, rabbits, guinea pigs, and dogs, they are mostly binucleated^56^. Although cardiomyocytes comprise the largest mass in the heart, fibroblasts, endothelial cells, and a neuronal mesh are also part of this organ^57^. In the case of innervation, its distribution in the heart is related to its function^58^. Recent studies have determined that sympathetic processes approximate the capillary to cardiomyocyte ratio and that in the rodent heart all myocytes interact with several varicosities of the same sympathetic process^57,59^. Sympathetic innervation of the heart consists of postganglionic fibers that enter the heart through the cardiac plexus from cervical and thoracic ganglia and that mostly interact with the atria and the base of the ventricles^58^. Parasympathetic postganglionic fibers emerge from cardiac ganglia diffusely located at the dorsal atrial surface of the heart and connected by a dense network of nerve fibers. Parasympathetic fibers are concentrated in sinoatrial and atrioventricular nodes, the atria, and the conducting system in the ventricles^58^. Sympathetic and parasympathetic nerves increase and decrease the heart rate, conduction velocity, and contraction force, respectively, by modulating the currents exchanged between cardiomyocytes and the intracellular Ca^2+^ concentrations that act on the actomyosin crossbridge cycle responsible for contraction^60^. Specifically, stimulated sympathetic nerves release norepinephrine that binds to β-adrenergic receptors, leading to the production of cyclic AMP, the phosphorylation of ion channels and other proteins, an increase in Ca^2+^ influx through the membrane and release from the sarcoplasmic reticulum, and greater depolarization, speed of contraction and heart rate^58^. On the other hand, activated parasympathetic fibers reduce contraction rates due to the interaction between released acetylcholine and muscarinic receptors^58^. This binding turns on a signaling pathway where G proteins produce the closing of Ca^2+^ channels and the opening of K^+^ channels and thus cause repolarization and decreased action potentials. Both the heart and innervation develop simultaneously due to reciprocal signaling, where cardiac-sourced cues influence neuronal survival, growth, patterning, and maturation while innervation has been related with the baroreflex, cardiomyocyte growth transitions, and the regulation of heart size during development^5,58,61^. Sympathetic and parasympathetic innervation have also been associated with the regenerative capacity of the neonatal mouse heart based on studies where physical and chemical nerve ablation impeded regeneration after injury^62,63^. Although heart transplantation remains the gold standard to treat cardiac failure, artificial cardiac pumps and total artificial hearts are also being developed^64^. Total artificial hearts are only for temporary use and serve as a “bridge to transplant”, helping the patient become fit to undergo transplantation^65^.

As an alternative, cardiac tissue engineering aims at replacing or repairing damaged cardiac tissue using biological or polymer-based scaffolds in combination with cells and signaling factors. Some of the most extensively used approaches towards developing tissue-engineered hearts include: (a) cultivation of scaffold-less 3D cellular stacks^66,67^, (b) repopulation of decellularized native tissue^68,69^, (c) mechanical stimulation of cells in hydrogels^70^, (d) cell cultivation on perfused channeled scaffolds^71^, (e) electrical stimulation of cells in porous scaffolds^72^, and (f) cell delivery in injectable hydrogels^73,74^. Although the importance of electrochemical cues in engineered cardiac tissue and the role of vascularization in the adequate functioning of implants has been explored, fabricating an artificial cardiac tissue or whole heart with appropriate innervation has been largely unexplored. Out of the few investigations considering this, human decellularized pericardial-derived scaffolds implanted in swine models were found to support neo-innervation after 30 days, as shown by the presence of myelinated and non-myelinated axons in the scaffolds^75^. In another case, given that neurotrophic factors produced by organs tend to drive innervation, engineered triple-layer cardiomyocyte sheets were transduced to overexpress glial cell-derived neurotrophic factor (GDNF) to promote innervation and then transplanted on cryoinjured rat hearts^76^. The overexpression of GDNF appeared to promote the presence of tyrosine hydroxylase+ (sympathetic) fibers in the sheets at earlier time points than controls, although no parasympathetic fibers were observed. An optimal scaffold for cardiac tissue engineering should either promote neo-innervation after implantation or be pre-innervated prior to implant to ensure functional recovery. On the latter point, research has been conducted on the in vitro co-culture of cardiomyocytes and sympathetic neurons, of murine and/or human sources, showing that these neurons can form synaptic-like connections with cardiac cells and tune their beating based on neuron-only pharmacological, electrical, or optical stimulation^77,78^. These findings may be applied to the formation of pre-innervated cardiac tissue, which could also be combined with pre-vascularization to create an even more clinically relevant cardiac patch^79^.

## Innervation in skeletal muscle tissue engineering

Skeletal muscles are striated, tubular, and multinucleated muscle fibers attached to the skeleton via tendons and under voluntary control of the SNS. The basic unit of contraction in skeletal muscle is the motor unit, composed of a somatic motor neuron that innervates multiple myofibers^80^. The neural input for muscle contraction originates in the primary cortex region (first order motor neurons) and travels through the corticospinal tracts to reach the ventral horn of the spinal cord that houses the second order motor neurons from which nerve fibers extend into the muscles. The signal is transmitted from the nerve terminal to specific myofibers by secretion of acetylcholine at the neuromuscular junctions (NMJs). This leads to depolarization of muscle fibers through Ca^2+^ influx and the generation of a muscle action potential. Although muscle function is primarily controlled by motor neurons, the role of sensory nerve endings as well as glial cells are increasingly being explored. Muscle proprioception and stretch reflex are mediated by mechanosensory nerve endings interacting with intrafusal muscle fibers within a muscle spindle as well as by Golgi tendon organs, which are proprioceptors located at the muscle-tendon junction innervated by afferent sensory fibers^81,82^. In addition, specialized glial cells called terminal/perisynaptic Schwann cells present in NMJs have a crucial role in preventing muscle fatigue by Ca^2+^ release and K^+^ uptake at the synaptic cleft through purinergic 2Y1 receptor protein^83,84^.

Severe musculoskeletal trauma like VML is associated with a frank loss of muscle as well as progressive motor axotomy, thereby necessitating tissue engineering strategies that can address these challenges^85,86^. Tissue-engineered skeletal muscle constructs have been fabricated using scaffold-based as well as scaffold-less technologies. Synthetic polymers and ECM proteins like collagen^87^ have been used as scaffolds, whereas scaffold-free techniques have involved self-assembly of skeletal muscle constructs using muscle stem cells (also called satellite cells)^88,89^ or tendon constructs^90^. Appropriate somato-motor innervation remains the outstanding challenge to fabricating a fully functional muscle^91^. A strategy to innervate muscle grafts or artificial tissues has involved surgically attaching them to host nerves. For example, engineered muscle constructs developed using self-assembly of primary myocytes have been connected to the sural nerve in vivo and have been reported to interface with this neural tissue^92,93^. Skeletal muscle tissue grafts generated via 3D bio-printing and surgically embedded with common peroneal nerve led to the formation of NMJs 2 weeks after implantation^94^. In addition, muscle grafts have been surgically inundated with multiple surrounding nerves (hyper-innervation)^95^. In another approach, embryonic motor neurons were injected into the distal tibial nerve stump one week after sciatic nerve transection. Regenerating axons were found to be myelinated and of smaller diameter forming simple NMJs. Intramuscular axon sprouting from transplanted neurons augmented muscle reinnervation, reduced atrophy, and restored muscle excitability^96^. In addition, we have employed the in vitro co-culture approach and developed innervated tissue-engineered muscles (InTEMs) composed of aligned neuromuscular bundles obtained by culturing spinal motor neurons with skeletal myocytes on aligned nanofibers^97^. Interestingly, these pre-innervated muscle constructs were found to promote myocyte fusion and maturation in vitro as well as augment muscle satellite cell migration, microvasculature presence, and NMJ formation near the injury following implantation in a rat model of VML^97^. Innervation has a crucial role in development, maturation and functional regulation of the musculoskeletal system and hence it may be imperative that tissue-engineered muscle be pre-innervated during construction and/or capable of robust innervation upon implantation^98,99^.

## Innervation in smooth muscle tissue engineering

Smooth muscles are spindle shaped, non-striated, uninucleated fibers lining the walls of internal organs, such as the bladder, intestine and trachea, and involved in involuntary control of bladder pressure, intestinal motility, airway passage and deglutition. The autonomic nerve fibers form varicosities when innervating smooth muscle fibers and release neurotransmitters within a wide cleft thereby forming a diffuse junction unlike the more spatially confined NMJs in skeletal muscles. The present article is restricted to discussing such innervation in the context of intestinal and bladder tissue engineering. The GI tract is extrinsically and intrinsically innervated. In the case of the former, preganglionic parasympathetic fibers coming from the dorsal motor nucleus of the vagus or the spinal cord, depending on the location of the tissue of interest, synapse with postganglionic neurons, such as myenteric neurons or other ganglia, within the esophagus, stomach, small intestine, and colon^4,100,101^. Preganglionic sympathetic fibers from the thoracic and lumbar spinal cord mostly interact with different prevertebral ganglia (e.g., celiac, superior mesenteric, inferior mesenteric, pelvic), from which postganglionic nerves act on myenteric and submucosal neurons, with only sparse innervation of the circular and longitudinal muscle layers of the GI tract^101^. The majority of sympathetic innervation in muscle regions is found in sphincters^4^. Sympathetic activity can inhibit GI muscle contraction and mucosal secretion, whereas parasympathetic activity can both activate and reduce these functions^101^. Smooth muscle contraction in the GI tract can occur by the release of acetylcholine from parasympathetic postganglionic neurons in the myenteric layer and the activation of muscarinic cholinergic receptors, while contraction can be inhibited by nitric oxide, vasoactive intestinal polypeptide, or purine release^102^. A similar cholinergic pathway is involved with the excitation of secretion from gastric parietal cells. Postganglionic sympathetic fibers regulate ENS activity and neurotransmitter release and GI immune function.

The ENS is the intrinsic nervous system component of the GI tract that innervates components of the gut wall with approximately 200–600 million neurons^4^. The ENS is formed by a myriad of ganglionic plexuses (i.e., myenteric and submucosal plexus) that regulate motility, secretion, and blood flow^39,103^. The myenteric plexus is a continuous circuit covering the entire GI tract, while the submucosal plexus is mainly observed in the small and large intestine^104^. Among ENS neurons there are intrinsic sensory neurons found in the myenteric and submucosal plexus and that are sensitive to mechanical distortion and the chemistry of luminal contents^4,104–107^. Excitatory and inhibitory neurons innervate the two muscle layers and the muscularis mucosae to modulate smooth muscle contraction and relaxation by the secretion of excitatory (e.g., acetylcholine, tachykinins) and inhibitory (e.g., nitric oxide, vasoactive intestinal peptide) neurotransmitters, respectively^4,108^. The mucosa is populated by secretomotor and secretomotor/vasodilator neurons that promote exocrine fluid secretion and increased blood flow in cholinergic and non-cholinergic varieties^4,104,109–112^. Various types of interneurons in the ENS participate in motility and secretomotor reflexes^4,104,113^. Common signaling pathways influence aspects of both ENS and gut development such as patterning, villi/crypt formation, stem cell and enteric neuron/glia proliferation and differentiation, cell cycle timing, neuron migration, and neurite fasciculation and directionality^100^. The relative importance of the intrinsic and extrinsic components of GI innervation depends on the organ. Intrinsic ENS innervation can independently control GI function, especially in the case of the small and large intestines with the exception of the rectum, as evidenced by several studies that showed non-significant morbidity effects after vagotomy and sympathectomy procedures^101,114–117^.

Intestinal tissue engineering is increasingly complex due to the presence of multiple cell types, apart from smooth muscle, the various functions that need to be supported, and the need for luminal flow^118^. Notably, the GI wall consists of an epithelial mucosa layer, submucosa, concentric longitudinal and circular smooth muscle layers, and a serosa. Most past attempts at GI tissue engineering and biofabrication have focused on harvesting tissue obtained from rodent^35,119^, swine^120^, or human^121^ intestines and seeding it in biodegradable scaffolds and on directing stem cells to become human intestinal organoids^122^, which lack innervation, as a more translatable approach. Innervation has been directly incorporated in smooth muscle sheets fabricated by coating aligned smooth muscle cells with enteric neural progenitors, and these tissues could be stimulated electrically and chemically and exhibited muscle and neuron-dependent contraction and relaxation^123,124^. In the case of co-cultures, light stimulation of human stem cell-sourced enteric neurons grown with human differentiated smooth muscle cells led to increased contractions, showing their functional integration^125^. Co-cultured smooth muscle cells also had enhanced maturation relative to controls based on a greater expression of mature markers and contractions after pharmacological stimulation. In the case of human intestinal organoids, these cell clusters have been innervated by aggregation with human neural crest cell (NCC)-derived ENS precursors, 3D culture in vitro, and maturation by engraftment in mice^37,126^. In another study, human pluripotent stem cell (PSC)-derived enteric NCC neurospheres were seeded with organoids onto a biodegradable scaffold and then implanted for 3 months in mice^126^. In both cases, the engineered tissue formed intestinal epithelia, smooth muscle layers, and plexuses proximal to the muscle and possessed a diverse neuronal population. The tissue also exhibited smooth muscle functionally integrated with the ENS as seen by neuron-dependent contractility resembling human intestinal motility^37^. Gene expression analyses also suggested that the presence of neurons, depending on the in vitro or in vivo environment, could alter genes related to GI development and promote fates of more proximal or distal parts of the GI tract^37,126^. In a more recent study, fibroblasts, mesoangioblast-derived smooth muscle, and NCC-derived neurons were cultured on decellularized rat esophagi in a bioreactor, allowed to mature and be vascularized for 1 week in vivo, and subsequently harvested and seeded with rat esophageal endothelial cells to create engineered esophageal tissue^127^. In all cases, the incorporation of the ENS was essential to creating constructs resembling native tissue in terms of structure and functionality.

The urinary bladder is innervated by sympathetic and parasympathetic nerves as well as branches of the SNS. Parasympathetic preganglionic neurons originate from the sacral segment of the spinal cord and interact with postganglionic neurons located in the detrusor wall and pelvic plexus. Axon terminals from the postganglionic neurons release acetylcholine and interact with muscarinic receptors present in bladder smooth muscles leading to bladder contraction^128^. The sympathetic pathway originates in the lower thoracic and upper lumbar spinal cord segments and takes a complex route into the inferior mesenteric ganglia ending in the pelvic plexus via the hypogastric nerves^129^. Postganglionic sympathetic fibers from the inferior mesenteric ganglion release norepinephrine and this leads to relaxation of the bladder wall^128^. Non-neuronal cells of the bladder, such as urothelial cells and myofibroblasts, interact with the local afferent and efferent nerves thereby regulating bladder function^130^. The urothelial cells constitute the inner lining of the bladder (urothelium) and express nicotinic, muscarinic, tachykinin, adrenergic, bradykinin, and transient-receptor-potential vanilloid receptors. Activation of these receptors through chemical stimulation or bladder movement can trigger release of mediators like ATP, nitric oxide, neuropeptides, acetylcholine and nerve growth factor (NGF) that can interact with adjacent nerves that then regulate bladder smooth muscle contraction/relaxation. Myofibroblasts are a unique population of cells present within the lamina propria of the bladder having cytological characteristics of smooth muscle cells and fibroblasts^131^. These specialized cells are in close proximity to unmyelinated axonal varicosities, express ATP-gated purinergic receptors, and possibly function as a “stretch receptor organ”^131,132^.

Urinary bladder tissue engineering has mainly focused on the reconstruction of the bladder wall employing acellular as well as cellular scaffold techniques^133–138^. Neural regulation of the bladder smooth muscle and urothelial layer is critical for proper functioning of the bladder. According to a recent review on the status of bladder tissue engineering, there are 141 published clinical studies using a variety of biomaterials^139^. Despite such extensive research on regenerating bladder wall components, there have been few studies that attempted to induce innervation and integration of tissue-engineered constructs with the host nervous system. Lack of innervation is one of the major challenges to commercialization of current therapies. One of the first studies to promote neural regeneration in tissue-engineered bladder constructs found that chitosan-based scaffolds induced innervation^140^. In order to achieve better innervation, the same group proposed the use of direct transplantation of Schwann cells to augment nerve regeneration into a damaged bladder wall^141^.

## Strategies for the incorporation/promotion of innervation in biofabricated constructs

Organ transplantation (e.g., heart, kidney, liver, lung) is the most prevalent surgical intervention for organ failure, but this procedure results in complete denervation of the organ. The process of reinnervation following transplantation can take days (renal reinnervation) to years (cardiac reinnervation)^142,143^. Although neurotization and targeted muscle reinnervation techniques (discussed below) have shown some success in functional regeneration of skeletal muscles, there are currently no strategies in clinical use to promote directed innervation in cardiac or smooth muscle tissue (with the exception of bladder). The process of reinnervation is generally left up to intrinsic, host-induced processes. It is also believed that vascular anastomosis of transplanted organs alone is sufficient to direct reinnervation due to inherent neurovascular association and functional interaction^144,145^. Despite this, such allogeneic vascular anastomosis often results in a chronic inflammatory condition, called cardiac allograft vasculopathy, after a heart transplant, which is a major impediment to long-term survival of the transplant^143,146,147^. Moreover, trauma and disease can also interfere with the nerve supply to each organ. Thus, there exists a need for more consistent strategies to ensure the integration of artificial organs with the host nervous system and the presence of nervous functional regulation, as well as to restore or promote regeneration of lost nerve connections in existing organs. Below we highlight potential avenues to achieve these goals for muscle tissue engineering based on both traditional and newer experimental methods.

### Surgical re-routing of adjacent host nerves

**Fig. 2:**
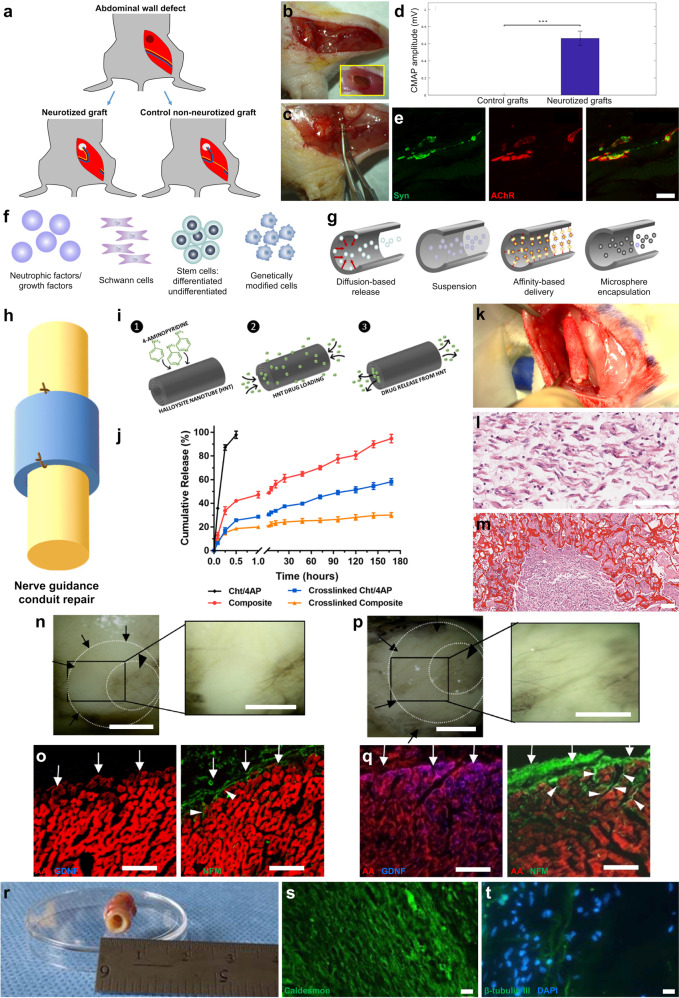
Strategies to promote innervation of biofabricated constructs. **a**–**e** Innervation of skeletal muscle grafts by neurotization. **a** Schematic of the repair of an abdominal wall defect in mice with a PLLA/PLGA porous scaffold seeded with myoblasts, endothelial cells, and fibroblasts. Neurotized grafts were innervated by suturing to the graft the proximal femoral bundle including the nerve. **b**, **c** Images of the neurotized and control grafts, respectively, with the white arrowheads indicating the grafts and the white arrows marking the femoral nerve. **d** Assessment of compound muscle action potential (CMAP) amplitude in the grafts after electrical stimulation of the attached femoral bundle. **e** Neuromuscular junctions in the grafts were stained for acetylcholine receptors (AChR; red) and synapses (synaptophysin; green). (Reprinted with permission from Kaufman and Kaplan et al.^152^; Copyright John Wiley & Sons). **f**, **g** Diagrams of examples of traditional strategies for promoting peripheral nerve regeneration using nerve guidance conduits modified to deliver neurotrophic/growth factors or cells using biomaterial-based delivery methods. (Reprinted with permission from Daly et al.^153^; Copyright The Royal Society). **h**–**m** Nerve guidance conduit functionalized for drug release to promote nerve regeneration**. h** Schematic of a nerve guidance conduit sutured to the proximal and distal ends of a damaged nerve to promote axon regrowth. **i** Halloysite nanotube (HNTs)-based conduits were loaded with 4-aminopyridine (4-AP), a potassium channel blocker that promotes neurotransmitter release and extends action potentials. **j** Conduits composed of drug-loaded chitosan, epichlorohydrin-crosslinked chitosan, chitosan/HNT composites, or crosslinked composites were analyzed for in vitro 4-AP release. Data presented as mean ± standard error of the mean. **k** Image of the repair of a rat sciatic nerve defect with a conduit. **l**, **m** Hematoxylin and eosin stain image of a longitudinal and cross-sectional view of the conduit, respectively, 4 weeks post repair showing regenerating nerve with infiltrating Schwann cells (conduit material in dark red). (Reprinted with permission from Manoukian et al.^158^; Copyright Elsevier). **n**–**q** Neurotrophic factor overexpression for the reinnervation of injured myocardium. Rat hearts denervated by cryoinjury were injected with adenoviruses encoding **n**, **o** GFP or **p**, **q** GDNF. After 5 days, the hearts were **n**, **p** whole-mount imaged for neurofilament-M (NFM) or **o**, **q** immunolabeled to denote cardiomyocytes (α-actinin (AA); red), GDNF (blue), and axons (NFM; green). Hearts with GFP overexpression showed only sparse axon presence, while GDNF overexpression led to a significant presence of axons in the injured area and axon growth into the myocardium. (Reprinted with permission from Miwa et al.^159^; open access, PLOS). **r**–**t** Engineered scaffolds with smooth muscle sheets co-cultured with neural progenitor cells for GI repair. **r** Image of the scaffold 14 days after subcutaneous implantation in the back of athymic rats. The scaffold consisted of human smooth muscle cells cultured on molds to promote alignment and human enteric neuronal progenitor cells added on top of the muscle sheets. The sheets were wrapped around a tubular scaffold of chitosan and collagen to resemble the structure of the gut. The engineered neuromuscular tissue was stained to show **s** contractile smooth muscle (caldesmon; green) and **t** differentiated neurons (β-tubulin III; green). (Reprinted with permission from Zakhem et al.^123^; Copyright Elsevier). Scale bars: **e** 10 µm; **l**, **m**, **s**, **t** 100 µm; **n**, **p** 1 mm; **o**, **q** 50 µm.

A nerve transfer or neurotization is a procedure in which surgeons reroute nerve pathways by transferring the end of a healthy nerve to the site of an injured nerve, with the goal of restoring movement or sensation. Such techniques are used in patients suffering from irreparable nerve injuries or amputation where residual nerves are transferred to reinnervate target muscles, which in the case of amputation can enable control of a motorized prosthetic device and also recovery of sensory feedback^148–150^. Similar nerve reconstruction strategies are also employed to achieve functional restoration in a decentralized bladder following SCI and have been extensively reviewed previously^151^. Surgical re-routing of adjacent host nerves toward an implanted tissue-engineered construct can be a potential method to achieve targeted innervation. Indeed, neurotization has been applied to innervate engineered muscle grafts fabricated by seeding a tri-culture of endothelial cells, fibroblasts, and myoblasts on porous scaffolds of poly-l-lactic acid (PLLA) and poly (lactic-co-glycolic acid) (PLGA)^152^. After myotube assembly in vitro for 7–14 days, the scaffolds were transplanted into an abdominal wall defect in mice and sutured to the isolated femoral nerve to provide innervation (Fig. 2a–c). The functionality of the muscle grafts was confirmed by stimulating the femoral nerve and analyzing compound muscle action potentials (CMAPs), which were only generated in neurotized grafts (Fig. 2d). Moreover, histological evidence of NMJs was observed in the grafts (Fig. 2e), serving as additional confirmation of innervation. Despite the need for more research into creating sophisticated and mature engineered tissues, neurotization as a strategy to promote graft innervation is aided by its well established nature in reconstructive surgery^152^.

### Scaffolds combined with biomolecule delivery

The innervation of engineered tissues and organs could also be accomplished by applying strategies traditionally used for peripheral nerve repair within biofabricated constructs. For example, the standard alternative to autografts is the use of nerve guidance conduits (NGCs) that provide a pathway for regenerating nerves to bridge defects. As reviewed elsewhere^153–156^, these biomaterial conduits may be hollow or have structural guidance cues to offer more support for cell migration and axon regeneration. An additional layer of improvement for NGCs has been addressing the neurotrophic and cell requirements for proper regeneration via inclusion of exogenous biomolecules or cells (Fig. 2f). Ideally, these constructs would feature controlled release of neurotrophins and neurotrophic factors that promote neural cell survival, growth, differentiation and migration, such as NGF, brain-derived neurotrophic factor (BDNF), neurotrophin-3 (NT-3), neurotrophin-4 (NT-4), GDNF, axon guidance ligands (e.g., netrins, semaphorins), and/or neuroprotective agents^153,154^. A critical aspect of biomolecule delivery involves tuning the spatiotemporal release based on the application, the specific molecule, and the mitigation of limitations related to stability, half-life, and side effects^153^. In this pursuit, these biomolecules may be delivered in suspension or contained within microspheres, bound to the conduit based on covalent crosslinking or affinity-based binding, or released by diffusion after degradation, among other methods (Fig. 2g)^153^. As an example, NGCs composed of chitosan and halloysite nanotubes (HNTs) were loaded with 4-aminopyridine (4-AP) by vacuuming a drug solution into the lumen of the nanotubes (Fig. 2h, i)^157,158^. This drug has been prescribed to multiple sclerosis patients and promotes nerve regeneration when administered exogenously^157,158^. These NGCs were shown to exhibit controllable burst and sustained release over time in vitro based on conduit composition and crosslinking (Fig. 2j) and to promote Schwann cell infiltration after implantation in a rat sciatic nerve defect (Fig. 2k, m)^158^. In another application more closely related to this manuscript, GDNF overexpression by viral delivery was shown to significantly promote sympathetic reinnervation of adult rat hearts after denervation when compared to controls (Fig. 2n–q)^159^. Axon guidance molecules are also a valuable strategy for conduit or scaffold functionalization given the pivotal role of axon pathfinding during development and regeneration following nerve injury^160,161^. All these approaches can be extended to engineered muscle tissues and scaffolds, which may be functionalized to deliver appropriate biomolecules that facilitate host innervation into these biofabricated tissues.

### Pre-innervation of tissue-engineered constructs employing anatomically inspired engineered axonal tracts

NGCs and other constructs can also be modified to incorporate neural cells associated with axon growth and regeneration processes to promote host innervation (Fig. 2f). These include Schwann cells that secrete neurotrophic factors, form aligned structures that guide regenerating axons (i.e., the Bands of Büngner), and participate in remyelination^153^. Cells may be genetically modified to overexpress certain neurotrophic factors, as mentioned previously with viral-based overexpression of GDNF. Similarly, engineered muscle tissues and organs can also be directly co-cultured with neural cells in order to be innervated prior to implant (Fig. 2r–t). Considering the crucial role of the physical presence of neurons/axons for the function, development, and maturation of muscle, it may prove to be essential that biofabricated tissues are pre-innervated appropriately (somatic/autonomic) during the construction process. We assert that most, if not all, engineered organs would benefit from a combination of embedded neurons and axonal tracts. The presence of appropriate neurons during the in vitro biofabrication process will ensure proper development, maturation, and functionality of tissue-specific cells. Aligned axon tracts in pre-innervated tissue-engineered organs can also act as “highways” that facilitate host innervation post-transplant in a targeted, directed manner. This hypothesis is based on our long experience with transplanting allogeneic aligned axonal networks and their ability to augment host reinnervation by providing topographical and biochemical cues to regenerating axons^13,16^. Such transplanted neurons and axonal tracts have been observed to be present months post-transplant in both rats and pigs, which would provide adequate time to guide host axons to reinnervate the biofabricated organ. A substrate for proper innervation post-implant would thus enable host-mediated functional regulation of the transplanted organs based on biological feedback in a self-contained manner.

In the following sections, we present our neural engineering technology and how it may be applied to create engineered neural tissue aimed at reconstructing damaged host nerve fibers, promoting host innervation, and incorporating these into other engineered tissues during fabrication in vitro for later implantation. We would like to emphasize that the concepts discussed in these sections are conceptual based on our experience with axon stretch growth technology (TENGs) and miniaturized 3D aligned axonal constructs (micro-TENNs) and is limited to the type and timing of innervation during biofabrication. Indeed, the biofabrication of innervated tissue-engineered muscle constructs demands careful consideration of biomaterial design and cell culture conditions like media composition and nutrient perfusion to address the challenges of culturing two or more different cell phenotypes. Although we do touch upon some of these challenges in this manuscript, detailed discussions on these aspects are beyond the scope of this article.

## Use of stretch-grown neural constructs for directed innervation of biofabricated tissues and organs

**Fig. 3:**
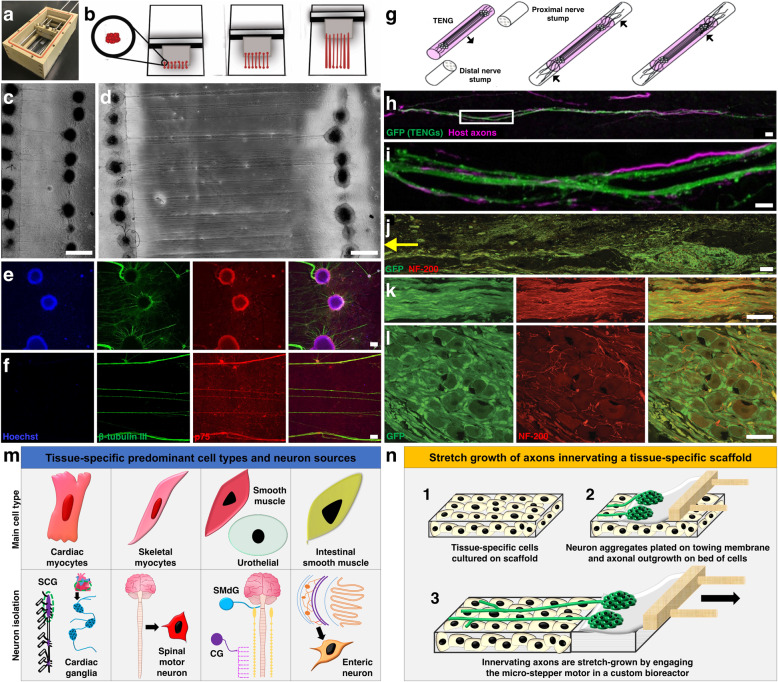
Stretch-grown nerve grafts and their application for the directed innervation of biofabricated constructs. **a** Stretch-grown tissue-engineered nerve grafts (TENGs) are fabricated employing a mechanobioreactor that has a towing membrane and a mobile towing block. **b** Aggregates of neurons are seeded on two sides of the towing membrane, allowed to grow connecting axons, and then separated at a specific rate by pulling the towing block to stretch the axons. **c**, **d** Phase contrast images of TENGs before and after application of mechanical forces to stretch the axon tracts. **e**, **f** Confocal images of aggregates and axon tracts, respectively, stained for nuclei (Hoechst; blue), axons (β-tubulin III; green), motor neurons (p75; red). (Reprinted with permission from Katiyar et al.^166^; authors of content, John Wiley & Sons). **g** TENGs are embedded in collagen and encased in a nerve conduit before implantation in a nerve injury model. (Reprinted with permission from Huang and Cullen et al.^13^; authors of content, Mary Ann Liebert, Inc.). **h**, **i** After implantation to repair a peripheral nerve lesion, host axons (SMI31; purple) were seen extending along the path dictated by TENG axons (GFP; green), thus demonstrating the mechanism of axon-facilitated axon regeneration. (Reprinted with permission from Struzyna et al.^16^; open access, Wolters Kluwer Medknow Publications). **j** Distal outgrowth of implanted TENG axons (GFP+) into the host nerve was observed 6 weeks post repair. Bundles of host axons (NF-200; red) projected along the construct and also into the distal nerve (yellow arrow). **k**, **l** Higher magnification images showing axonal bundles and ganglia in the implanted TENGs, respectively. (Reprinted with permission from Huang and Cullen et al.^13^; authors of content, Mary Ann Liebert, Inc.). **m** Stretch-grown axons may be incorporated into tissue-specific biofabricated constructs by first obtaining the main cell types and neurons associated with each type of tissue. Abbreviations: superior cervical ganglia (SCG), celiac ganglia (CG), submandibular ganglia (SMdG). **n** (1) After creating a scaffold seeded with tissue-specific cells, (2) neurons can be mechanically aggregated by centrifugation, plated on the towing membrane and cultured to ensure attachment of their neurites to cells in the scaffold. (3) Axons projected from the aggregates would be stretch-grown by controlling the rate at which the towing membrane is pulled away, thus creating a construct with directed innervation that could be used for in vitro or in vivo applications. Scale bars: **c**, **d** 1000 µm; **e**, **f** 500 µm; **h** 25 µm; **i** 6 µm; **j** 100 µm; **k**, **l** 25 µm.

During development, physical stretching of axons, the majority of which occurs after axons have reached synaptic targets, is driven by expansion of the body (e.g., bone growth in the periphery, gray matter/cell layer expansion in the brain), resulting in axonal tracts spanning several centimeters in the brain and up to one meter in the periphery to innervate distal end targets^14,162,163^. Based on seminal discoveries by Smith et al.^14^, this developmental process for controlled and rapid axon growth has been recapitulated in vitro using continuous mechanical tension applied within custom-built mechanobioreactors. This work was initially performed using sensory neurons/axons^13,15,164,165^, but our group recently expanded this technique to include motor neurons/axons^166^. Here, our strategy to biofabricate TENGs involves the seeding of two populations of neuronal aggregates within a custom mechanobioreactor that enables controlling the rate of growth of the axons projected across the aggregates (Fig. 3a, b). Upon reaching the desired axon length using an escalating rate of stretch growth (generally 1–5 mm/day, but rates of up to 1.0 cm/day have been achieved), the formed axonal tracts are embedded in a collagenous matrix and then rolled into a long cylindrical form to create TENGs. Thus, TENGs are living 3D nerve constructs that consist of longitudinally aligned axonal tracts spanning discrete neuronal populations, thus mimicking aspects of the structure of the lost nerve (Fig. 3c–f)^14^. For transplantation into PNI models, TENGs are inserted within a NGC for subsequent suture into a nerve defect (Fig. 3g). TENGs were found to be capable of repairing nerve defects and driving host axon regeneration along their length by providing topographical and biochemical cues to regenerating axons based on the newly discovered mechanism of axon-facilitated axon regeneration or “AFAR” (Fig. 3h–l)^13,16,165^. We anticipate that TENGs fabricated with appropriate autonomic/somatic neurons can be employed to project axons into target cells cultured within bioreactors in vitro and/or target tissue in vivo. This fabrication method may mimic the native architecture of axonal networks that travel long distances from autonomic ganglia to end organ targets. In this section, we will propose a potential paradigm to employ our TENG technology to include stretch-grown axons during the biofabrication of tissues and organs using neurons representative of the anatomical locations of native innervation and the main cell types in each tissue (Fig. 3m). Overall, for all muscle tissues discussed in this review, the main cell type can be cultured on an appropriate biomaterial scaffold chosen according to the application. After sufficient growth and maturation, the seeded scaffold can further develop within a mechanobioreactor, whereby sympathetic and/or parasympathetic neuron aggregates are allowed to extend axons and attach to the scaffold, and then mechanical stretch is employed to produce biofabricated muscles innervated by long, aligned axon tracts generated via “stretch-growth” (Fig. 3n).

### Pre-innervated cardiac muscle

In order to fabricate an innervated engineered myocardium, it is necessary to select the appropriate cell types, scaffolds, and culture conditions for construct maturation. Cardiac myocytes, the principal cell type in the heart can be isolated from embryonic or neonatal mice, adult rats, or human myocardium following established protocols^167–170^ and cultured on scaffolds for 3–5 days^171,172^. As shown in Fig. 1 and Fig. 3m, the intracardiac ganglia are an appropriate source for parasympathetic neurons, whereas the sympathetic population can be harvested from superior cervical ganglia (SCG). There are established protocols for the isolation of intrinsic cardiac ganglia^173^ and the SCG^172,174,175^ from prenatal or postnatal mice or rats, while sympathetic neurons can also be obtained from the directed differentiation of human PSCs^77,176^. As the postganglionic parasympathetic pathway is embedded within the heart projecting from intracardiac ganglia, neurons isolated from these ganglia can be directly co-cultured with cardiac myocytes growing on a scaffold. The scaffold containing cardiac myocytes and intracardiac ganglia neurons can then be housed within a mechanobioreactor to allow stretch growth of aligned sympathetic axons on the surface. This may mimic the anatomy of postganglionic sympathetic nerves projected to the heart from distant sympathetic ganglia. Although cardiomyocytes comprise most of the cardiac tissue, these are not the only cell types present. The heart also includes fibroblasts, endothelial cells, and pacemaker cells, which have a critical role in cardiac function^177,178^. Hence, in order to accurately recapitulate cardiac physiology in an engineered heart, future studies should be directed towards culturing multiple types of cardiac cells along with proper innervation.

### Pre-innervated skeletal muscle

Motor and sensory neurons originate within (motor) or immediately adjacent to (sensory) the spinal cord and travel long distances to innervate skeletal muscles. Consequently, stretch-grown motor axons mimic physiological axon growth mechanisms and structure^164^. Although skeletal muscles are served by both sensory and motor branches, the initial biofabrication strategy might be restricted to the generation of motor axon-innervated skeletal muscles since regaining motor function is often the priority for patients suffering from neuromuscular trauma. In cases with significant neuromuscular damage/loss like VML, the ideal surgical intervention would entail fabrication and implantation of bioengineered nerve-muscle complexes. There are numerous reports describing the fabrication of NMJs in vitro through co-culture of motor neurons and skeletal myocytes^179–181^. However, such co-cultures do not have sufficient biomass for implantation to repair/replace injured neuromuscular tissue. As previously mentioned and shown in Fig. 3c–f, our research group has developed TENGs using spinal motor neurons^166^. In order to generate implantable neuromuscular constructs with anatomical relevance, we propose to stretch-grow engineered motor neuron aggregates on a bed of pre-differentiated myofibers grown on a suitable substrate^166^. Motor neurons can be harvested from rodent spinal cords^182^ or obtained from differentiated human PSCs^183^ using published protocols, while skeletal muscle cells can be sourced from cell lines, rodents^184^, or human PSCs^185^. This concept would entail culturing the skeletal muscle cells on a scaffold for 4–7 days in differentiation media to allow for the formation of myofibers prior to introduction and mechanical stretch of motor neurons in mechanobioreactors. Following the formation of neuromuscular connections, the construct may be allowed to mature in a bioreactor to form an “off-the-shelf” implantable nerve-muscle complex with the appropriate biomass.

### Pre-innervated smooth muscle

Smooth muscle tissue in organs like the urinary bladder have sympathetic and parasympathetic innervation wherein axons travel from their source ganglia to the target tissue. This phenomenon may be simulated by stretch-growing a mixed population of sympathetic and parasympathetic ganglia using our mechanobioreactors. A readily available parasympathetic cell source for culture and for biofabrication of innervated bladder appears to be the submandibular ganglia^186^, although it is not necessarily anatomically relevant. The pelvic ganglia would be the ideal choice for sympathetic neurons^187^; however, its status as a sympathetic source is a recent finding and hence not well established. Thus, the celiac ganglia may be a better established source for the culture of sympathetic neurons^188,189^ for biofabrication of pre-innervated bladder tissue if using an animal source (Fig. 3m). Engineering a functional bladder would require culture of urothelial cells along with the bladder smooth muscle cells, which can be harvested from different layers of bladder tissue from rodent, porcine, or human sources^190–194^. Moreover, both urothelial cells^195,196^ and bladder smooth muscle cells^197,198^ can be induced from human PSCs. An underlying layer of bladder smooth muscle cells would facilitate growth and maturation of urothelial cells^190,191^. This can be achieved by initial culture of bladder smooth muscle cells on a scaffold followed by plating of urothelial cells to mimic the native architecture of the bladder wall. Although the bladder has autonomic and somatic control, we have restricted our strategy to the autonomic pathway considering it has a more important role in bladder development. Based on the timing of innervation during development (within a few weeks of conception in humans)^199–203^, we anticipate that the autonomic neurons should be introduced early (within 2–3 days) in culture and stretch-grown on the surface of the seeded scaffold to ensure proper maturation of the bladder tissue in vitro. Extensive studies are necessary to design the appropriate biomaterial scaffolds to accurately mimic the intricate structure of bladder tissue.

The GI tract has its own neural population (i.e., the enteric neurons) that has a more predominant role in its development and function relative to its parasympathetic and sympathetic innervation. As anatomically the enteric neurons lie within the intestinal wall layers, we do not anticipate the need to apply stretch growth. As such, for ENS-innervated intestinal tissue constructs the ideal strategy would involve co-culturing intestinal smooth muscle cells and enteric neurons in 3D scaffolds. Smooth muscle cells may be harvested from murine or human tissue^123,204^, and enteric neurons can be derived from precursor cells^37^ or from primary culture of the myenteric plexus^205^. Previous studies have successfully created tubular scaffolds consisting of human smooth muscle sheets cultured with human enteric neural progenitors^123,206^. These scaffolds exhibited a contractile smooth muscle phenotype, positive staining for neuronal markers, and proper functionality based on electrical field stimulation and force generation changes in response to exposure to acetylcholine and vasoactive intestinal peptide. As the culture conditions for innervated intestinal constructs have been explored, future research should be aimed at producing more clinically translatable and complex intestinal tissues recapitulating the multiple cell types present in the layers of the GI wall in addition to muscle and neurons (e.g., intestinal epithelial cells, glia, vasculature, lymphatic cells). As previously mentioned, some researchers have produced human intestinal organoids with a functional ENS, which also contain other epithelial and mesenchymal cell types involved in GI function^37^.

## Micro-tissue engineered neural networks as a tool for localized axon delivery

**Fig. 4:**
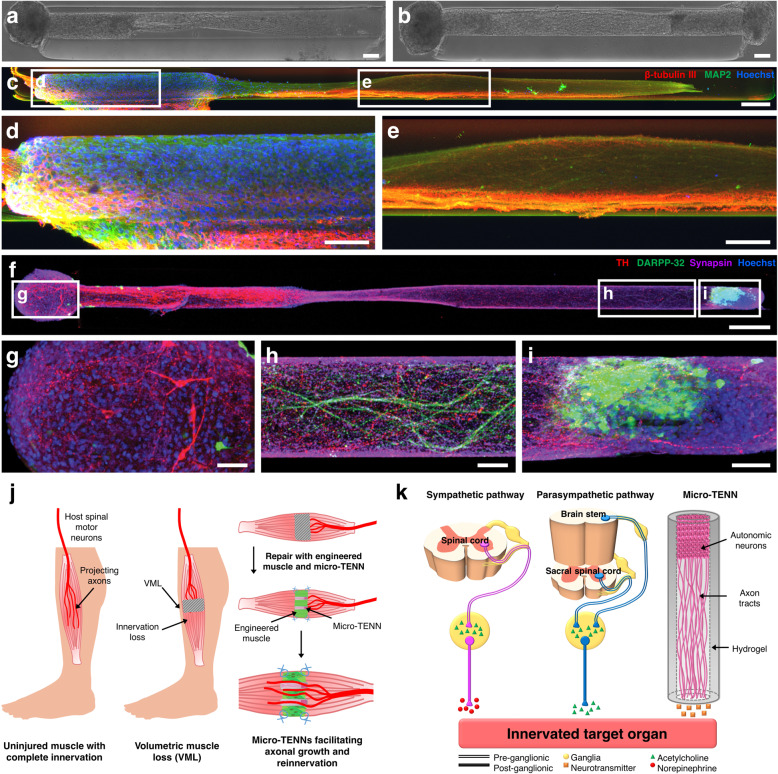
Application of micro-tissue engineered neural networks (micro-TENNs) for peripheral reinnervation. **a**, **b** Phase contrast images of a unidirectional and bidirectional micro-TENN at 5 days, respectively, consisting of an aggregate of rat embryonic cortical neurons seeded at one (unidirectional) or both (bidirectional) ends of a hydrogel micro-column. These aggregates extend aligned axonal tracts throughout the extracellular matrix-filled lumen. **c** Confocal image of a micro-TENN with neurons from the cerebral cortex at 28 days and stained for axons (β-tubulin III; red), somata/dendrites (MAP2; green), and nuclei (Hoechst; blue). **d**, **e** High magnification images of the aggregate and axon tract regions of the micro-TENN construct, respectively. (Reprinted with permission from Serruya et al.^210^; authors of content, John Wiley and Sons). **f** Bidirectional micro-TENN at 14 days fabricated with an aggregate of rat embryonic ventral midbrain neurons (left) and striatal neurons (right) and stained for dopaminergic neurons (tyrosine hydroxylase (TH); red), medium spiny (striatal) neurons (DARPP-32; green), synapses (synapsin I; purple), and nuclei (Hoechst; blue). **g**–**i** Zoom-ins show the dopaminergic aggregate, outgrowth from medium spiny neurons, and physical integration of dopaminergic axons with the striatal target, respectively. (Reprinted with permission from Struzyna et al.^21^; authors of content, Copyright John Wiley and Sons). **j** Micro-TENNs may serve to promote regeneration of axonal connections from spinal motor neurons to muscles suffering volumetric muscle loss (VML). In this application, micro-TENNs can be microinjected at the proximal nerve and with engineered muscle distally to guide axon growth from the nerve back to the muscle belly to regenerate lost neuromuscular connections. **k** Micro-TENNs could be sourced from autonomic ganglia to serve as parallel pathways to native autonomic innervation that could project axons to innervate the target organs. Scale bars: **a**, **b** 100 µm; **c** 200 µm; **d**, **e** 100 µm; **f** 250 µm; **g** 50 µm.

In a different fabrication process, we have created engineered constructs called micro-TENNs that consist of an aggregate of neurons seeded within the collagen-filled lumen of a hydrogel micro-column (345–710 μm in diameter) and projecting millimeter- to centimeter-scale aligned axonal tracts (Fig. 4a, b)^19,21,207–210^. These micro-TENNs have been traditionally designed for minimally invasive implantation in the CNS featuring neurons isolated from the cerebral cortex (Fig. 4c–e) or the ventral midbrain (Fig. 4f–i) for applications in cortical–thalamic pathway reconstruction^208^ and as synaptic-based interfaces with cortical circuitry^210,211^, or as replacements for the degenerated nigrostriatal pathway in Parkinson’s disease^21^, respectively. These constructs replicate the structure of axonal pathways projecting from a discrete neuronal population, and thus may also mimic the ganglia–axon tract architecture in the PNS/ANS. These constructs represent a unique platform for localized, minimally invasive delivery of living neurons with preformed, directionally constrained axon tracts to facilitate host axon ingrowth and guidance as well as act as an exogenous neural interface to connect with peripheral organs and modulate their activity. In addition, micro-TENNs allow co-culture of at least two aggregates, each with a different cell phenotype (neuronal/non-neuronal), which may be useful to elucidate how autonomic/somatic neurons interact with tissue-specific cells.

### Somatic neuron-based micro-TENNs for the preservation of the motor end plate of distal muscles

In severe cases of muscle injury like VML, there is damage to intramuscular nerves as well as loss of connection between the injured muscle and nerve. A pre-innervated tissue-engineered muscle could be a potential strategy to replace/repair the damaged muscle. However, a large loss of muscle volume followed by surgical intervention may increase the risk of damage to the main nerve connecting with the muscle belly. We anticipate that our micro-TENN technology can be used to fabricate miniaturized columns of aligned axons that can be used to “re-wire” fine branches of the main nerve with the injured or tissue-engineered muscle (Fig. 4j). This would not only help in promoting axonal growth across the micro-TENN and implanted engineered muscle but also prevent prolonged periods of denervation and thereby preserving the motor end plates.

### Autonomic neuron-based micro-TENNs for interfacing with peripheral organ/tissues

Taking the micro-TENN technology forward, our team is also developing artificial constructs composed of autonomic ganglia and their projected axonal tracts to interface with target muscle tissue in a spatiotemporal manner based on synaptic communication and host feedback. These engineered constructs would act as living parallel pathways mimicking the form and function of the sympathetic and parasympathetic ganglia/fibers that naturally innervate/modulate organs (Fig. 4k). Our strategy has the advantage of being highly specific, as a set of artificial autonomic axon constructs may be created for each organ-of-interest to provide both sympathetic and parasympathetic control. Micro-TENNs as interfaces could be applied as exogenous sources of ANS axonal inputs to supplement and/or replace endogenous inputs in cases of trauma or disease. In addition, employing a controllable and neuron-specific stimulation paradigm (e.g., optical, magnetic, near infrared) can make micro-TENNs a biologically inspired alternative for the modulation of peripheral organ function. This is in contrast to common vagal nerve stimulation techniques, which would not only affect the organ of interest, but many other targets as well, and only provides the ability for parasympathetic modulation.

## Challenges and advancements towards fabricating innervated tissue-engineered constructs

Despite the exciting promise of cross-disciplinary tissue engineering for the purposes outlined in this article, the road to achieving complex engineered tissues and complete artificial organs is extremely challenging. The use of neurotization, biomolecule delivery, co-culture with neural cells, and the incorporation of stretch-grown and aligned axonal tracts represents only generalized strategies for promoting the innervation of biofabricated muscle constructs. In reality and evidently, different types of tissues and organs offer their unique set of challenges apart from the need for innervation. Flat and tubular tissues have been engineered with more success because of their simpler structures and cell content^212^. On the other hand, hollow/viscus and solid organs require more sophisticated tissue-engineered strategies in order to be replicated given their much greater metabolic demands, variety of functions, need for extensive vascularization, architectural complexity, multiplicity of cell types and interactions, and difficulty in emulating their developmental processes in vitro^212,213^. Vascularization has been a longstanding subject of research in the field because of its close relationship with the scalability of engineered constructs. More advanced 3D printing technologies have been developed in past years to enable precise control of the distribution of cells, biomaterials, and biochemical guidance cues within a complex architecture matching the tissue of interest and offering ways to ensure proper nutrient and oxygen transfer^94,214^. Moreover, vascularization and innervation are intricately linked to one another. This is especially true for sympathetic innervation, which closely follows blood vessels. For example, venous endothelin has been found to guide sympathetic innervation in developing hearts^215^. Similarly, neurotrophic factors like NGF, BDNF, GDNF, and NT-3 have been reported to enhance angiogenesis in different tissues like skin, heart, and cartilage through receptor-mediated activation or recruitment of proangiogenic precursor cells^215,216^. Spinal motor neurons secrete BDNF whereas astrocytes can express a range of neurotrophic factors^217^. Further, sensory nerves influence the branching pattern of blood vessels in the skin through secretion of vascular endothelial growth factor (VEGF)^144^.

The issue of limited and immunologically compatible cell sources has also been a constant shortcoming. However, this issue has received a push forward from the generation of tissue-specific and/or neural-specific cells from human PSCs, particularly patient-sourced human induced pluripotent stem cells (iPSCs)^218^. Human iPSCs can augment the translatability of tissue-engineered constructs by ideally representing a potentially personalized and unlimited source of immunocompatible cells of any phenotype. In addition, using these human cells would move tissue-engineered constructs using rodent-derived neurons or tissue-specific cell types beyond being only models into being applied in clinical settings. Many of the cell sources highlighted in this review for muscle constructs have been derived from human iPSCs in previous studies. For example, cardiac myocytes^219^, skeletal muscle tissue^185^, bladder smooth muscle cells^197^, and intestinal organoids^220^ have been derived from human iPSC sources. In addition, iPSC lines have been used to generate most of the neuronal cells that would be needed to innervate engineered tissues. Sympathetic neurons have been differentiated using the activation of Wnt, Sonic hedgehog and bone morphogenetic protein signaling, and culture with media including NGF, GDNF, BDNF, and ascorbic acid^77^. Approximately 40–60% of differentiated cells expressed peripherin, an intermediate filament in peripheral neurons, and tyrosine hydroxylase and dopamine β-hydroxylase, enzymes involved in norepinephrine synthesis. These neurons were also functional, responding to stimulation by exhibiting action potentials and releasing catecholamines in a Ca^2+^ and Na^2+^ channel-dependent manner. Notably, these neurons physically integrated with cardiomyocytes in co-culture and modulated their beating rates based on pharmacological and light stimulation. A differentiation protocol published in a different study reported a greater yield of sympathetic neurons of 75–80%, which shows the variability of these processes^176^. In terms of motor neurons for skeletal muscle engineering, several studies have reported the induction of motor neurons from iPSCs, as reviewed elsewhere^183^. For example, a recent protocol involved promoting Wnt signaling, inhibiting Notch signaling, adding retinoic acid, activating hedgehog signaling, and subsequent exposure to ascorbic acid, CNTF, BDNF, NT-3, and GDNF^221^. Differentiated motor neurons after 21 days expressed the neuronal markers β-tubulin III and microtubule-associated protein 2 (MAP2) and the motor neuron markers choline acetyltransferase (ChAT) and homeobox HB9, with 73% showing HB9. In terms of functionality, the neurons were able to exhibit synchronized action potentials in connection with other neurons based on calcium activity recordings. On the other hand, human iPSCs have been differentiated by activation of Wnt signaling and exposure to retinoic acid into vagal NCC progenitors, the main source of the ENS after migrating to colonize the bowel^37,125,222^. After 15 days, enteric NCC progenitors can be induced to become enteric neurons with media containing GDNF and ascorbic acid^222^. These cells can become cholinergic, GABAergic, serotonergic, and nitric oxide-producing neurons like those found in the ENS, as confirmed by immunostaining and flow cytometry after 50–75 days^125,222^. The differentiated enteric neurons could also functionally integrate with smooth muscle cells by modulating their contractions in response to neuronal stimulation with light^125^. The precursors have also differentiated into a functional ENS after implantation with intestinal organoids into the mouse kidney capsule^37^ and migrated along and repopulated the mouse colon^125^. Interestingly, co-culture of human sympathetic and enteric neurons with cardiomyocytes and smooth muscle cells, respectively, seemed to promote neuronal maturation as well^77,125^. This aspect suggests that pre-innervation of engineered tissues could also be beneficial for the neurons themselves.

In spite of the widespread attention on iPSCs, there are several limitations if applying these cells for tissue engineering purposes. Specifically in the case of neurons, we did not find any protocol for deriving a parasympathetic population from an iPSC source, which highlights a limitation for using these cells for engineered tissues and an area still requiring more research. In a practical manner, differentiation of human iPSCs requires technical expertise and may involve long protocols (typically around or more than 30 days) to obtain cells with a desired purity or maturity. Each independent batch of iPSCs may have distinct differentiation efficiencies and may need to be carefully evaluated for consistent pluripotency, gene expression, and functionality^222^. The effectiveness of iPSC differentiation into the desired cell phenotypes may not be reliable, jeopardizing the homogeneity and functionality of obtained cells for further use^223,224^. The presence of contaminating cell types may be a factor limiting the viability and durability of cultures^222^. Obtaining mature differentiated cells is also inconsistent and may be hampered by them being mostly in an embryonic or fetal stage, which may be a crucial limitation for having fully functional innervated engineered tissues for implantation^77^. There may be issues with tumor-forming aberrant behavior of iPSC-derived cells^223^. Moreover, there need to be more expansive assessments of the degree to which differentiated cells recapitulate human tissue in vivo in terms of marker expression, morphology, and function^183,222^. Improvements and standardization of stem cell culture protocols aimed at reproducing the 3D environment, cell-cell interactions, and the cell lineages present during normal organogenesis, apart from the presentation of differentiation factors, may represent an approach to solving these issues^223,224^.

Culturing stem cells in 3D and considering the presence of various cell phenotypes, in the hope of creating more physiologically and structurally relevant human 3D tissues, is precisely at the core of the organoid approach. In this strategy, stem cells are differentiated into multiple cell types that self-assemble into 3D clusters, called organoids, exhibiting organ-like structures and function and the capacity for self-renewal^225,226^. Organoids have been explored as potential components of tissue replacement therapy; indeed, organoids have been developed to simulate aspects of the heart^227,228^, bladder urothelium^229,230^, skeletal muscle^231^, and GI tract^232–236^. Multilineage tissue engineering combining organoids and neural cells can also serve to model the feasibility of the functional integration between neurons/axon tracts and biofabricated tissues. Despite their wide-ranging applications in regenerative medicine and as research tools, organoids alone resemble tissues only at a small scale and are limited by inconsistent differentiation, maturation, organization, shape, and viability relative to each other even in the same culture^225,226,237^. Thus, challenges remain to achieve the scale up required for clinical translation. As one example to address this challenge, we have recently developed micro-TENNs using human iPSC-derived organoids, which were able to produce centimeter-scale bundled human axon tracts within an implantable hydrogel column^207^. The field still requires more knowledge about organogenesis and the complex interactions that guide the assembly of organoids^237^. Moreover, organoids generally lack other cell types present in vivo (e.g., stromal, immune, neural cells)^226,237^. Improvements should be made to the signaling factors and ECM components presented to them in order to more realistically capture the in vivo microenvironment^226^. These strategies, in addition to co-culturing with other cell types and employing tissue engineering, will be at the forefront of making organoids even more representative of native tissues and organs.

Notwithstanding this set of challenges shared broadly across virtually all applications of tissue engineering, proper innervation is in itself an important consideration when analyzing challenges and limitations. A crucial aspect is ensuring that the engineered tissue has the correct proportion of neuronal cells/fibers and main tissue cell types (e.g., cardiomyocytes, skeletal myocytes, smooth muscle cells) and the appropriate ratio of different types of innervation (e.g., parasympathetic, sympathetic, sensory, enteric phenotypes). Because of the tightly regulated relationship between muscle function and innervation, providing incorrect innervation may lead to aberrant tissue and organ function or localized neuroma resulting in detrimental effects for patients. For example, aberrant sympathetic and parasympathetic innervation of cardiac tissue can result in ventricular tachyarrhythmia or atrial fibrillation, which can lead to sudden cardiac death^238–240^. Incorrect autonomic innervation of the bladder can cause neurogenic bladder and the development of symptoms akin to urofacial syndrome, which is a congenital disease characterized by urinary incontinence and incomplete bladder emptying^241,242^. Similarly, implanting motor axon laden constructs in a skeletal muscle tissue could lead to polyinnervation and spontaneous muscle fibrillation.

Another crucial aspect involves the functionality of pre-innervated engineered tissues and organs, for which it is imperative to evaluate the precision of neural control during fabrication as well as post-implant in a clinical scenario. Due to current advancements in optogenetics and neuro-electronics, it is possible to study innervation of cells, tissues and organs using electrical, chemical, or optical methods of neural stimulation. Sympathetic control of cardiomyocyte contractile activity in culture has been assessed upon pharmacological (nicotine) and optogenetic stimulation (using channelrhodopsin-expressing neurons)^77^. Similarly, the formation of functional NMJs in skeletal muscle and motor neuron co-cultures can be evaluated upon chemical (l-glutamate), electrical, or optical (using light-responsive neurons) stimulation of the motor neurons and measuring contraction force or electrical output from twitching myofibers^243^. Neural regulation of cellular activity can also be validated using standard blockers of action potential. For example, in the biofabrication of intestinal tissue, tetrodotoxin (TTX) has been used to inhibit neuronal activity to ascertain the effect of neurons on the contractility of smooth muscle^126^. In preclinical in vivo studies, the neural control of tissues or organs has been generally assessed by electrical stimulation of the major nerve innervating the region of interest. Functional reinnervation of bladder was evaluated in canine models of SCI by electrical stimulation of the pelvic plexus followed by monitoring detrusor muscle contraction and increase in bladder pressure^244,245^. In musculoskeletal trauma-related studies, reinnervation of injured muscle was assessed by measuring isometric muscle contraction force upon neural stimulation along with monitoring changes in gait pattern over time^246^. One of the major challenges towards exploring innervation in tissue-engineered grafts is the lack of standardized methods to reliably assess the extent and quality of reinnervation in clinical scenarios. This is especially true for heart transplant patients, where most of the methods for evaluating autonomic innervation of cardiac muscle involve invasive techniques like determining catecholamine levels following an intracoronary injection of tyramine^143^. Less invasive techniques for evaluating sympathetic reinnervation involve tracking catecholamine radioisotopes like ^123^I-meta-iodo-benzylguanidine (MIBG) and ^11^C-hydroxyephedrine (11C-HED) using positron emission tomography (PET)^247,248^. Vagal parasympathetic reinnervation after heart transplant can be assessed directly using PET tracers^249^ or indirectly by measuring heart rate variability and monitoring its high frequency power spectrum^44,250^.

In terms of our TENG and micro-TENN “living scaffolds”, these technologies allow for the fabrication of spatially segregated, phenotypically controlled axonal constructs but still pose significant challenges towards being applied as clinically translatable sources of innervation for engineered tissues and organs. For example, TENGs are the only technology that allows continuous axonal stretch to generate long aligned axonal tracts. However, stretch-induced axonal growth is yet to be explored in the context of parasympathetic and sympathetic neuronal populations. This is a major challenge since the ability to withstand mechanical forces varies among different cell types, which implies the need for extensive optimization of stretch growth parameters. In addition, the stretch growth technology is not high throughput at present and will possibly require weeks to months to generate constructs at sufficient lengths to mimic the lost anatomy. The concept of stretch-grown pre-innervated tissue-engineered muscle constructs discussed here also does not take into account the complex fascicular structure of nerves as well as the high degree of arborization presented by some nerves closer to the nerve-muscle interface. On the other hand, micro-TENNs have the unique advantage of being injectable as well as suturable into individual fascicles or fine branches. Although micro-TENNs are an exciting platform for potential neuromodulation of organs, we have yet to validate the functional integration of micro-TENNs with peripheral nerves and their capacity to modulate host circuitry based on external inputs. The current generation of micro-TENNs has only been tested by implantation in rats and, as a result, these constructs need to be scaled-up for the large diameters and lengths required for larger animal models or humans. Moreover, even though we have shown the creation of micro-TENNs with human embryonic stem cell-derived dopaminergic neurons^21^ and stretch growth of human cortical neurons has been reported^251^, more work is needed to optimize both micro-TENNs and TENGs for the use of human autonomic neurons and to ensure clinical relevance. Innovative biomaterial encasement strategies and neurosurgical approaches will be required to implant pre-innervated constructs with their attached TENGs or micro-TENNs as a complete assembly and to “wire-in” these axonal tracts to the host nerves.

## Conclusions

### Innervation is crucial for proper organ integration and biofeedback

Innervation is necessary to provide a means for nervous system control and regulation of tissues and organs. Although most transplanted organs function independently of PNS/CNS inputs, nerves are involved in biofeedback and precisely regulating the functions of these organs generally via the ANS. Innervation may also be crucial to enable post-transplant integration and, if necessary, remodeling and/or functional refinement based on precise physiological needs of the recipient. To innervate engineered organs, host axons must be precisely driven to appropriate location(s) within the engineered organ, often over long distances. Therefore, neural tissue engineering and/or axon guidance strategies are a necessary adjunct to most organogenesis endeavors across multiple tissue and organ systems.

### Axon-based “living scaffolds” for the creation and integration of biofabricated organs

Although the role of innervation in organ development and function is fairly well established, its implementation during biofabrication of artificial tissues and organs remains a challenge. As axons are the primary mediators of crucial developmental cues, efforts to co-fabricate organs with neurons/axons may have an important role in ensuring proper structure and function of engineered replacement organs. Our team is actively building axon-based “living scaffolds” that may physically wire in and/or serve as a substrate to effectively drive targeted long-distance growth of host axons. This key technology may be utilized broadly in the field as an adjunct for general tissue and organ engineering efforts, as well as living bridges upon implant to facilitate innervation with the host nervous system. Therefore, many endeavors to create engineered organs may benefit from embedded axon-based scaffolds to ensure proper development/function and to facilitate host innervation post-transplant.

### Development of artificial organs requires a multifaceted approach

The ultimate goal of engineering complete artificial tissues and organs is still an extremely daunting task that will require extensive collaboration between the fields of stem cell biology, developmental biology, 3D biofabrication, biomaterials, and regenerative medicine. Based on the current state of the art, we anticipate that stem cell-derived, multilineage constructs (including neural phenotypes) created with complex cellular structures and guidance patterns will be a cutting-edge approach towards achieving this goal. Although we recognize that innervation alone will not push the field towards building clinically relevant artificial tissues, we emphasize the crucial role that innervation should have in engineered tissue constructs and organs for transplantation or as biofidelic test platforms. Moving forward, the tissue engineering field would be wise to devote more attention to integrating innervation into various processes and thus form the necessary multidisciplinary teams to properly execute this essential component in the genesis of tissues and organs.

## Data Availability

No new datasets were generated or analyzed for this manuscript. Citations for reproduced images/data are included.
